# Asynchronous Synchronization: A Spatially Explicit Agent‐Based Model Simulating *Ficus* Trees and Their Obligate Wasp Pollinator

**DOI:** 10.1002/ece3.73778

**Published:** 2026-06-10

**Authors:** Michael Palace

**Affiliations:** ^1^ Earth Systems Research Center University of New Hampshire Durham New Hampshire USA; ^2^ Department of Earth Sciences University of New Hampshire Durham New Hampshire USA

## Abstract

The study of figs (*Ficus* spp., Moraceae) has received considerable attention in the scientific literature, due to the genus' large number of species (700), pollination mutualism with Agaonid wasps, and its role as a keystone food resource in tropical ecosystems. Temporal sexual separation in hermaphroditic *Ficus* and asynchronous flowering among individuals within populations creates problems in supporting a viable population of dependent pollinator wasp species. To maintain the short‐lived wasp populations, *Ficus* populations must provide a continuous temporal sequence of flowering trees, which are havens for the pollinating, termed the Critical Population Size (CPS). CPS was defined at the threshold between parameter settings yielding no wasps at the end of any simulation run and those in which at least one tree retained wasps for all 100 model runs for a setting within a scenario. A theoretical model of fig–wasp persistence dynamics incorporating temporal and spatial components was developed to examine CPS in monoecious species. Parameterization of this model is from literature and applicable to many species of *Ficus*. Because male and female fig flowers occur sequentially within a tree, the model represents the timing of the male and female phases separately rather than combining them into a single flowering period. Distance wasps can fly is essential in sustainable populations of *Ficus*. The influence of other model parameters on the system and how the system responds when longer simulation times are conducted. The model developed here can examine the transfer of pollen and catalog the links between trees, essentially allowing the examination of a network that varies temporally but not spatially. This model develops a new concept on what is deemed a viable pollinating population and includes spatial attributes and the ability to track individual trees and wasps.

## Graphical abstract

Normal or baseline scenario with parameterization from the literature. The green line indicates the CPS based on the decision tree. The blue hatched area indicates the transition period of the model where there are increasing number of wasps present at the end of the simulations and an indication that the model is becoming more stable.

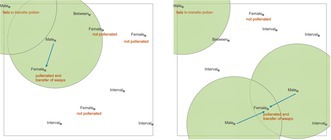

## 1 Introduction

Tropical forests throughout the world are undergoing rapid conversion and alterations due to human influence (Davidson et al. 2012). Impacts of land change impact biotic functioning and ecosystems not only through conversion to cropland and pasture, but also through the forest fragmentation, impacting vegetation dynamics as well as influences on animal populations (Bush et al. 2015). The interactions between tropical forest flora and fauna are complex and interconnected, such as mutualistic relationships between flowering plants and pollinating insects (Bronstein et al. 2006; Aguiar et al. 2024). As tropical forests are further impacted and fragmented by humanity it is vital to understand systematic relationships of the flora and fauna (Harrison 2005; Mello et al. 2021; Boyle et al. 2025).

One genus of tropical plants that has received considerable attention in the scientific literature is figs (*Ficus* spp. Moraceae) (Nason et al. 1998; Berg 1989). The interest in *Ficus* is due to the genus' large number of species (700), pollination mutualism with Agaonid wasps, and its role as a keystone food resource in tropical ecosystems (Ramirez‐Benavides 1970; Bronstein and McKey 1989; McKey 1989). Hermaphroditic *Ficus* undergo temporal sexual separation and asynchronous flowering to support a viable population of dependent pollinator wasp species (Gates and Nason 2012). In dioecious species, female and male reproductive functions are separated between individual trees (Miao et al. 2023). However, in monoecious species, female and male reproductive functions are contained in the same individual fig tree (Bronstein and McKey 1989). Some fig species show strong dichogamy, meaning the male and female flower phases occur one after the other within the same plant. In monoecious trees, female and the male reproductive phases are separated from 3 to 20 weeks, going from a male to a female phase (Bronstein and Patel 1992).

Temporal sexual separation in hermaphroditic *Ficus* and asynchronous flowering among individuals within populations create problems in supporting a viable population of dependent pollinator wasp species (Bronstein et al. 1990). To maintain the short‐lived wasp populations, *Ficus* populations must provide a continuous temporal sequence of flowering trees, which are havens for pollinating wasps and egg layer centers (Ghara and Borges 2010). To have a continuous temporal sequence of flowering trees it has been suggested that there is a minimum number of trees, termed the Critical Population Size (CPS) (Bronstein et al. 1990; Kameyama et al. 1999). Continued deforestation and fragmentation of the continuous forest in the tropics may lead to local wasp extinctions and loss of a viable population of *Ficus* (Mawdsley et al. 1998; Cottee‐Jones et al. 2016). Loss of a viable population of such a keystone resource would have ramifications for fauna populations such as birds and monkeys (Phillips 1997; Matthews et al. 2014). One way to explore such a system is through ecological modeling (Kameyama et al. 1999).

There are many types of models used in ecological science (Jackson et al. 2000; Milligan and Rohde 2024). One group of models used in environmental studies are Agent Based Models (ABM). ABMs are powerful tools for analyzing complex systems characterized by non‐linearity, heterogeneity, feedback, thresholds, resilience, and emergent behavior (Galvin et al. 2006; Rouchier 2017; Sabzian et al. 2018). They simulate system dynamics from the bottom up by modeling the actions and interactions of individual agents. Each agent follows a set of programmed rules or algorithms, often informed by empirical data and shaped by the environmental conditions and interactions they experience (Parker et al. 2008). An ABM was developed for this study with individual trees acting as individual agents and wasps as the interacting feedback that synchronizes a pollinator network.

In this study a temporal and spatial model was developed to examine CPS utilizing concepts from Bronstein et al. (1990). It is emphasized that the model presented here is a theoretical framework rather than a species‐specific reconstruction of any single fig–wasp system. Parameter values are drawn from empirically studied systems in the literature to ensure biological realism in magnitude and structure, but they are not intended to represent a coevolved pair or any actual ecological association. Instead, this approach allows exploration of general mechanistic constraints governing persistence in fig–wasp mutualisms.

While some species have both male and female phases within a single plant, this study focuses on species with male and female phases separated among individuals. This model is unique because it examines population density of *Ficus* and the importance of wasp flight distance, allowing for analysis of temporal and spatial dynamics across the landscape. The model was parameterized using literature that is applicable to many species of *Ficus*. One concept explored is that the distance of wasp flight is essential in designing sustainable populations of *Ficus*. This model also examines the transfer of pollen and the links between trees, essentially allowing the examination of pollination networks. Three questions are explored in this paper: (1) What is the CPS with different parameterization and initial conditions? (2) How does the network change with a set number of trees with varying flowering and flight distance parameters? and (3). How does varying the length of simulation change the connection between trees?

## 2 Methods

### 2.1 Model Structure

**FIGURE 1:**
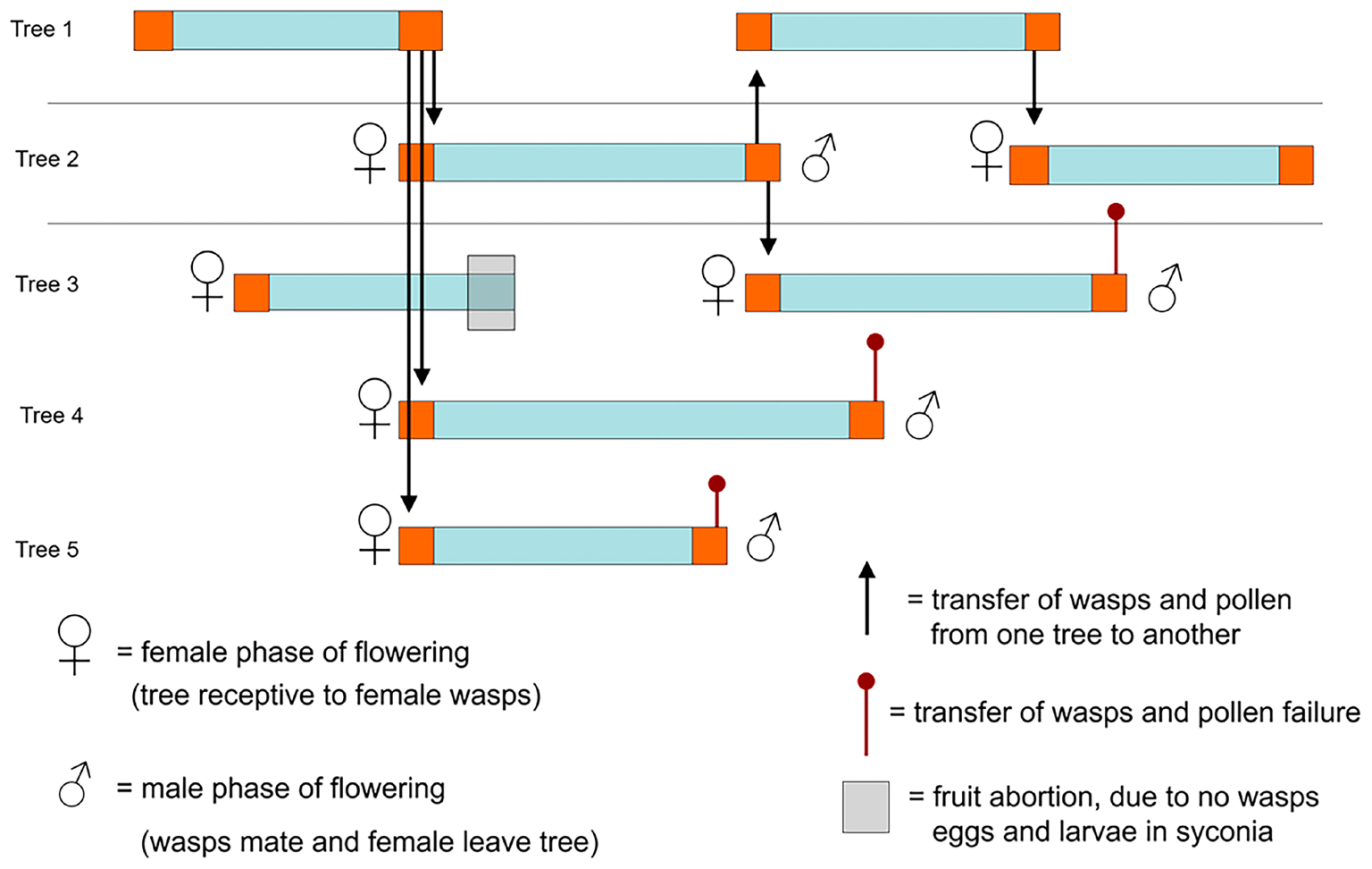
A schematic of the model, based on Bronstein et al. (1990). This schematic does not include the spatial component. This model shows the four phases a tree progresses through, allowing for the maintenance of a wasp population.

**FIGURE 2:**
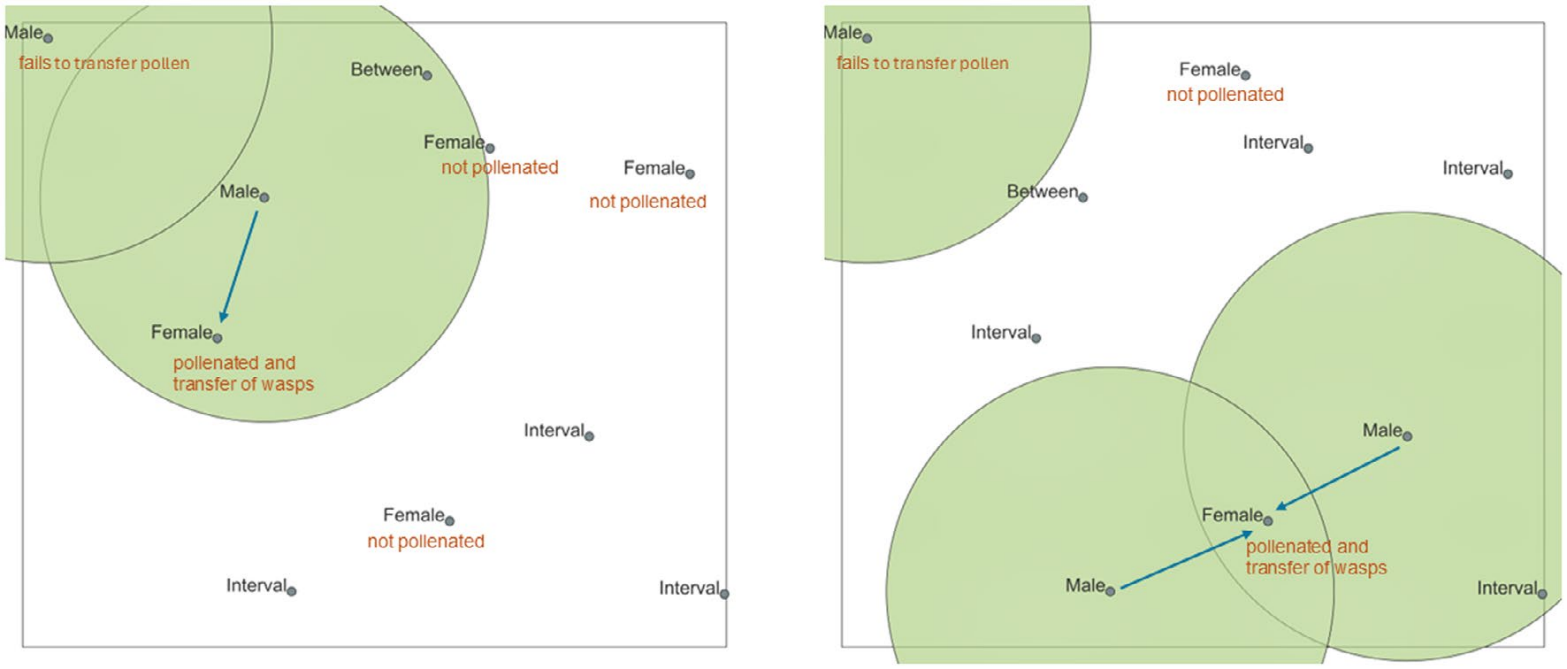
Spatial component of the model indicating distance a wasp can fly and the phases of flowering (male and female), interval between flowering, and duration (time between female and male flowering phases). Two time steps are shown.

The model presented in this paper is based on concepts from Bronstein et al. (1990) with a schematic of the non‐spatial model presented in Figure 1. The parameterization used in this study is intentionally synthetic and theoretical. Specifically, flowering phenology parameters are derived from *Ficus natalensis* (Bronstein et al. 1990), while fig wasp dispersal distance is based on estimates from a pollinator associated with *Ficus dugandii* (Nason et al. 1998). It should be noted that these two fig species occur in different geographic regions. These values are not intended to represent a coevolved species pair; rather, they are drawn from well‐characterized empirical systems to ground the model in biologically realistic ranges while enabling exploration of general dynamical behavior across fig–wasp systems. Parameters (mean and variance) of the phenological stages are presented in Table 1. A spatially explicit example of the model is presented in Figure 2.

**TABLE 1:** Parameters of the agent‐based model. Flowering phenology is from *Ficus natalensis* (Bronstein et al. 1990), and wasp flight distance is from the wasp associated with *Ficus dugandii* (Nason et al. 1998).

Flowering parameters	Weeks
Interval between successive flowerings (weeks)	22
Variance in interval	12.4
Duration of reproductive episode (weeks)	1.3
Variance in duration	0.2
Male phase	1
Female phase	1

Spatial components	Distance
Spatial dimensions of run	25 km × 25 km
Wasp flight distance	14 km

### 2.2 Model Structure

The model simulates the spatiotemporal dynamics of tree phenology and wasp movement using a discrete, daily time step. Each tree has a unique parameterization that can be changed at any time during a simulation.

### 2.3 Initialization

The initial location of each tree varies is randomly assigned *x* and *y* coordinates for each model run. Tree locations in the model are assigned randomly across the simulated landscape, without imposing explicit spatial structure such as patchiness, corridors, or edge effects. In this formulation, the landscape can be interpreted as a set of geospatially distributed trees that may represent a fragmented region, but without prescribed habitat configuration. This random placement is used to generate a broad ensemble of spatial configurations across many simulations, allowing general patterns of persistence to be assessed across heterogeneous but unstructured landscapes.

Each tree is assigned an initial phenological phase as well. To parameterize the model, standard normal random variates were generated using the Box–Muller transform for parameters that require a specified mean and variance (Box and Muller 1958). The length of each tree's phenological stage varies as well, following a normal distribution based on the mean and variance. Initially, trees are randomly assigned to a phenological stage as well as the time along such a flowering sequence (Table 1).

All trees in the simulation begin with a wasp population in all trees. Initializing all trees with wasps ensures that early model dynamics are not dominated by stochastic colonization effects and that observed patterns reflect endogenous dispersal and phenological processes rather than initial condition artifacts. The model was then run for a sufficiently long duration to allow these initial conditions to dissipate, ensuring that estimates of the population size required to maintain a viable wasp population were not deterministically influenced by the initial state of the model.

### 2.4 Temporal Progression

At each daily time step, trees currently in the male phase are identified as potential sources of dispersing female wasps. For each dispersal event, the distance between male‐phase trees and female‐receptive trees is calculated. The model was run for 208 weeks (approximately 4 years), consistent with previous modeling studies (Bronstein et al. 1990), and extended simulations were also conducted up to 40 years (2080 weeks) to evaluate long‐term dynamics. Across all simulations, results were found to stabilize early in the run, with initial adjustment dynamics dissipating well before the end of the simulation period. No separate burn‐in period was explicitly removed, as model behavior became consistent across time once initial conditions equilibrated.

### 2.5 Distance Calculation

The Euclidean distance between two trees is computed using the Pythagorean theorem.

For a source tree *i* located at coordinates $\left(x_{i},y_{i}\right)$ and a target tree *j* at $\left(x_{j},y_{j}\right)$, the distance $d_{\mathrm{ij}}$ is given by:

$$d_{\mathrm{ij}}=\sqrt{{\left(x_{i}-x_{j}\right)}^{2}+{\left(y_{i}-y_{j}\right)}^{2}}$$

A dispersal event is permitted when $d_{\mathrm{ij}}$ is less than or equal to the maximum wasp flight distance. If the distance between a source and a receptive tree is less than the maximum wasp flight distance, female wasps are transferred to the receptive tree, and the dispersal event is recorded as a link between trees. Following dispersal, all trees age by 1 day, and trees advance to the next phenological stage if the duration of their current stage has been reached.

### 2.6 Phenological Stage Transitions

When the end of a phenological cycle is reached, the next state is calculated using average and variance parameter values found in Table 1. Some figs exhibit pronounced dichogamy, with male and female floral phases occurring sequentially within the same individual, and for modeling purposes this temporally separated phenology was represented as a unified flowering sequence, female phase, interval, male phase. Again, the Box‐Muller transform was used to determine a standard normal distribution used in model parameterization (Box and Muller 1958). Phenological cycles vary each time a tree goes to a new phase; thus, a tree going into the interval between flowering phases for a second time has a new time in phase calculated for it. When a tree enters a new male or female reproductive stage, the stage duration is fixed at 7 days. If no wasps are present in a tree, the phenological cycle does not move into the male phase and the fruit crop is aborted; and the tree moves into the interval phase. This rule shortens the overall phenological cycle and prevents the occurrence of male phases in the absence of wasps. Because each tree has its own parameterization, sets its own flowering sequence, and records the storage of past links to other trees, the model is essentially an ABM. This allows for unique parameterization for each tree or the flexibility to change phenological time or stages for individual trees.

The model assumes that the presence of at least one surviving wasp is sufficient to establish a pollination link, thereby treating pollination success as a threshold (presence–absence) process rather than a continuous function of pollinator abundance. This simplifying assumption allows focus on persistence dynamics, but does not explicitly represent density‐dependent variation in pollination probability.

### 2.7 Model Scenarios

The tree population was incrementally increased by one from 10 to 500 to examine how population size influences CPS. Each scenario was run with a range of tree numbers (490) and run for 100 times, or 49,000 model runs per scenario. The minimum length of time, 208 weeks (4 years), has been used in previous models to estimate CPS and was used here. The model was also run for 208, 416, 832, and 2080 weeks (40 years), in an effort to examine how links in the network and synchronization might develop or change over time. This is deemed a scenario when all runs used the same parameters, but the tree population number varied. This allowed for the examination of the number of aborted fruit crops, the number of trees with wasps at the end of the simulation, and the minimum number of wasps at the end of the 100 runs. The threshold for CPS was defined as the transition between parameter settings in which no wasps were present at the end of any simulation run and those in which at least one tree retained wasps at the end of every run. This was deemed a failure rate in the system in which any of the runs had no wasps present in the system at the end of the simulation run.

Several scenarios were conducted to examine how parameter changes influence CPS, number of crop abortions, and number of wasps present at the end of the simulation, and these are presented in Table 2. These included changes in flower variations, number of trees, and length of simulation. The length of simulation and number of trees were also examined to see the influence on the number of links between trees in a simulation run.

**TABLE 2:** Overview of scenario simulations, accompanying descriptions, and decision‐tree (partition model) outputs for estimated critical population size (CPS).

Model run	CPS	*r* ^2^
Normal	245	0.779
All distance	237	0.766
Half distance	315	0.778
Half flower variance	276	0.732
Twice flower variance	282	0.704
Half flower time	307	0.686
Twice flower time	239	0.744

A decision tree was used because it does not assume linearity and is well suited to identifying threshold responses and nonlinear transitions between population persistence and collapse. Simulation outcomes were analyzed using a decision tree, which splits the data based on the predictor and split value that maximizes differences in predicted outcomes. This approach was used to estimate the minimum wasp population size required for persistence. The decision tree was fit to end‐of‐simulation minimum wasp abundance across model runs and was used to identify threshold population sizes separating persistence from collapse. Model performance was assessed using *R* ^2^.

The model was written in MS Visual Basic 4.0. Data was output as a comma delimited file. Figures were developed using SigmaPlot 10. Data organization, summary of scenario runs, and statistical analysis were done using JMP Pro 18.0.2. Additional figures were developed using QGIS 3.40, specifically for the spatial model representation. Network graphs, power law figures, and Kolmogorov–Smirnov and Zipf tests (Newman 2005) were performed using Python 3.9.7.

## 3 Results

**FIGURE 3:**
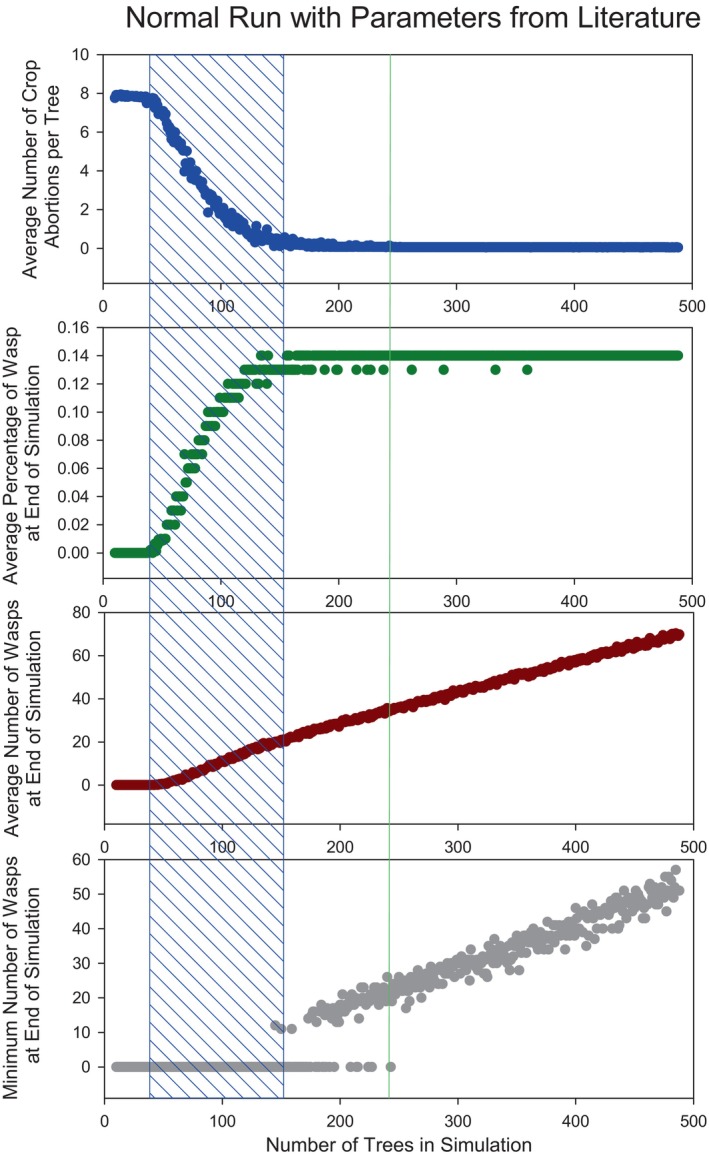
Normal or baseline scenario with parameterization from literature. The green line indicates the CPS based on the decision tree. The blue hatched area indicates the transition period of the model where there are increasing number of wasps present at the end of the simulations and an indication that the model is becoming more stable. CPS was determined to be 245.

A baseline simulation scenario with parameters from Table 1 is presented in Figure 3. Each simulation was conducted with a fixed number of trees and repeated 100 times. For each scenario, the average final wasp abundance was reported, along with the minimum number of wasps observed at the end of a simulation. The lowest final wasp count across all 100 runs was also identified, and this minimum value was used to determine the CPS. The CPS was estimated by applying a Decision Tree (partition) model to the final wasp counts across all scenarios. This approach allowed us to isolate the specific “tipping points” in the model that consistently resulted in a minimum viable population. The CPS was estimated to be 245 trees based on flowering phenology from *Ficus natalensis* (Bronstein et al. 1990) and wasp flight distance associated with *Ficus dugandii* (Nason et al. 1998). It was also found that the pollination network was in a transitional state to about 150 trees by examining both the percentage of trees with wasps present for runs with varying tree numbers. In addition, the number of abortions per tree in simulations levels out at 235 trees.

**FIGURE 4:**
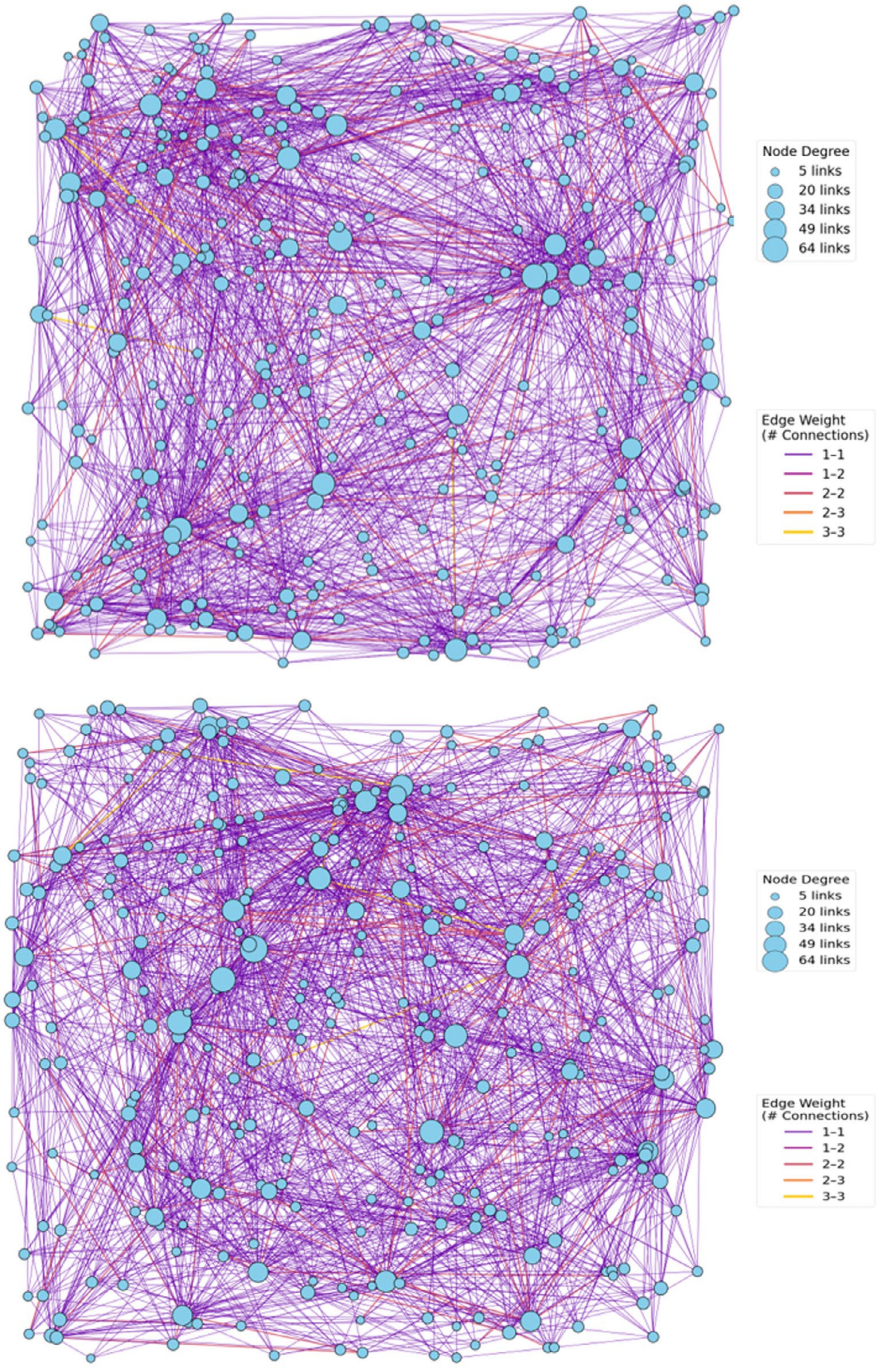
Example of two model runs with 300 trees simulated for 208 weeks for the baseline simulation. Node size is based on the number of links a tree has over the simulation. Edge weight is the number of times two trees were connected through the pollinating wasps. The landscape dimension was 25 km × 25 km.

The network with trees as nodes and pollination links between trees as edges is presented for two model runs in the baseline scenario developed in Figure 4. These runs were conducted with 300 trees and run for 208 weeks (4 years). Network figures were also developed for two model runs, each with 3000 trees and run for 2080 weeks (40 years) (Figure S1).

**FIGURE 5:**
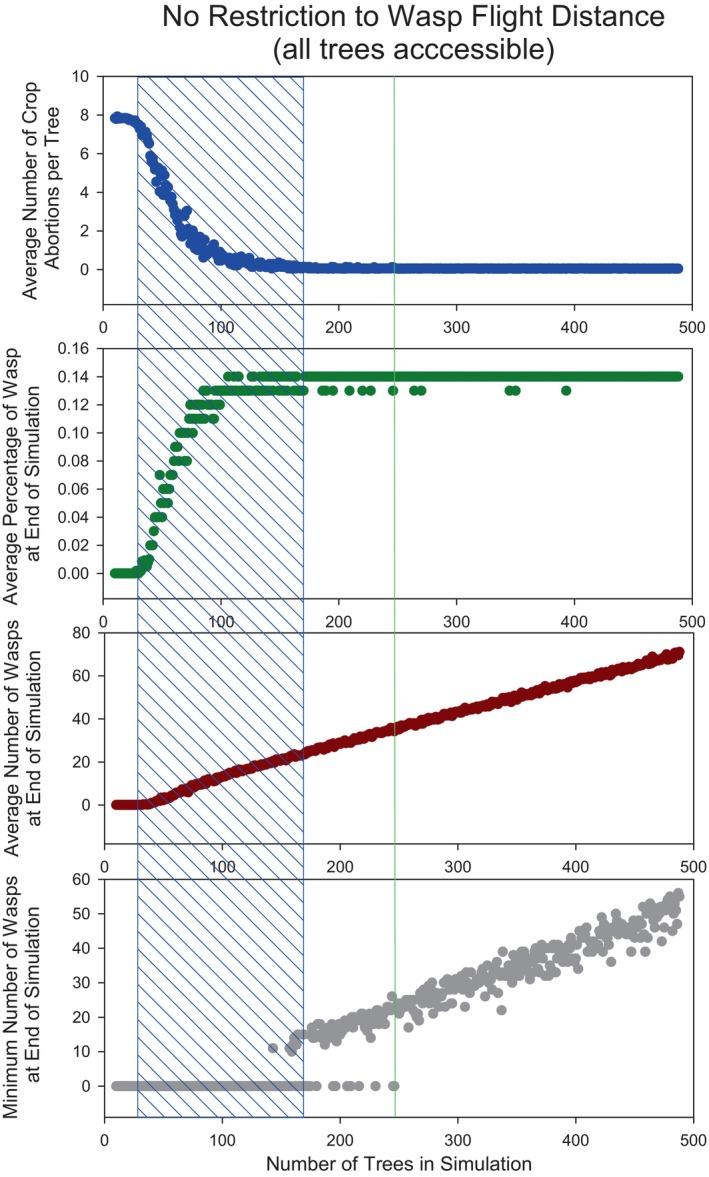
Simulation runs with no restriction to the distance for wasp flight. The green line indicates the CPS based on the decision tree. The blue hatched area indicates the transition period of the model where there are increasing number of wasps present at the end of the simulations and an indication that the model is becoming more stable. CPS was determined to be 237.

For the second scenario, the flight distance was changed so wasps could access all trees in the landscape, thus there were no restrictions on flight distance (Figure 5). It was found that the number of trees for CPS for this scenario was 237. There was a minor increase in the average number of wasps at the end of the simulation and a reduction of abortions per tree. The third scenario was conducted where the flight of wasps was reduced by half the baseline distance, to that of 7 km. Using the minimum number of wasps as an indicator of CPS, the number of trees was determined to be 315. The model was in transitional states up to 325 trees. There were still instances of no wasps present at the end of the simulation time with half the wasp flight distance at larger tree numbers.

Two scenarios were conducted that modified the flower variance, or the variability of time between male and female flowering stages. The scenario where the variance was cut in half resulted in a CPS of 276 with the transitional state of the model being much closer to the CPS. In the scenario where there was an increase in the flower variance that was twice that in our baseline scenario, the CPS was reduced to 282 and the transitional state was very close to the CPS.

Two scenarios were also run where the male and female flowering times were reduced by half or doubled from the baseline scenario. When the flowers were open for half the time, the CPS increased to 307 and the transitional state of the model was up to about 250. When the flowers were open for 2 weeks, or twice the baseline parameter, the CPS was reduced to 239 and the transitional state of the model was close to the CPS.

**FIGURE 6:**
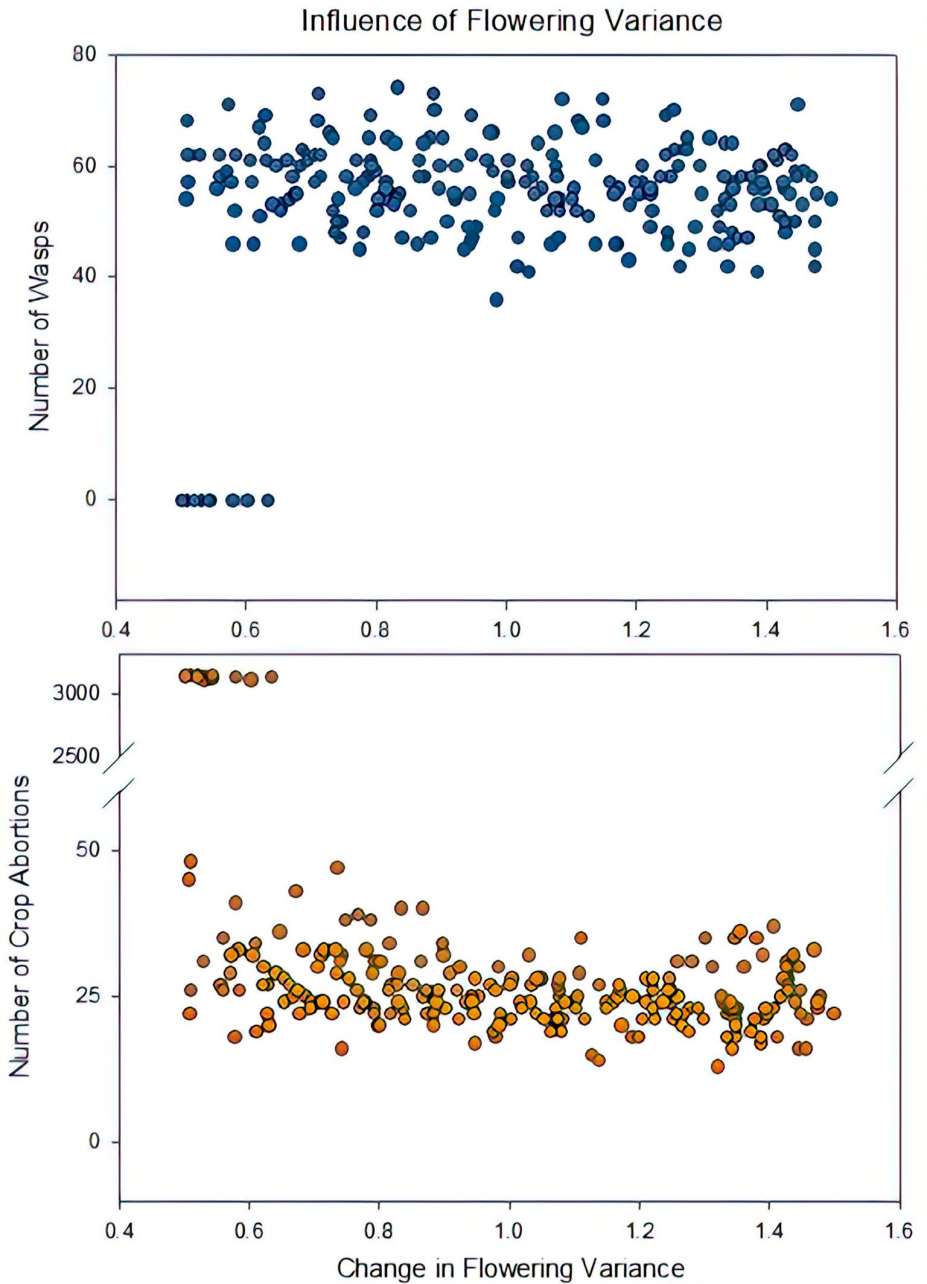
Sensitivity analysis examining how changes in flower variance influence the number of wasps present at the end of the simulation and the number of abortions.

A sensitivity analysis was conducted with normal baseline settings for all other parameters but one varying specific parameter in a sensitivity analysis. All runs were conducted 100 times. First, the flowering variance was from 0.5 to 1.5 and the influence on the number of wasps and the number of crop abortions (Figure 6). There was very little variation across the results except for values below 0.65, indicating that though flowering variance is important when its value is very low, once the model network is functioning, there is little influence.

**FIGURE 7:**
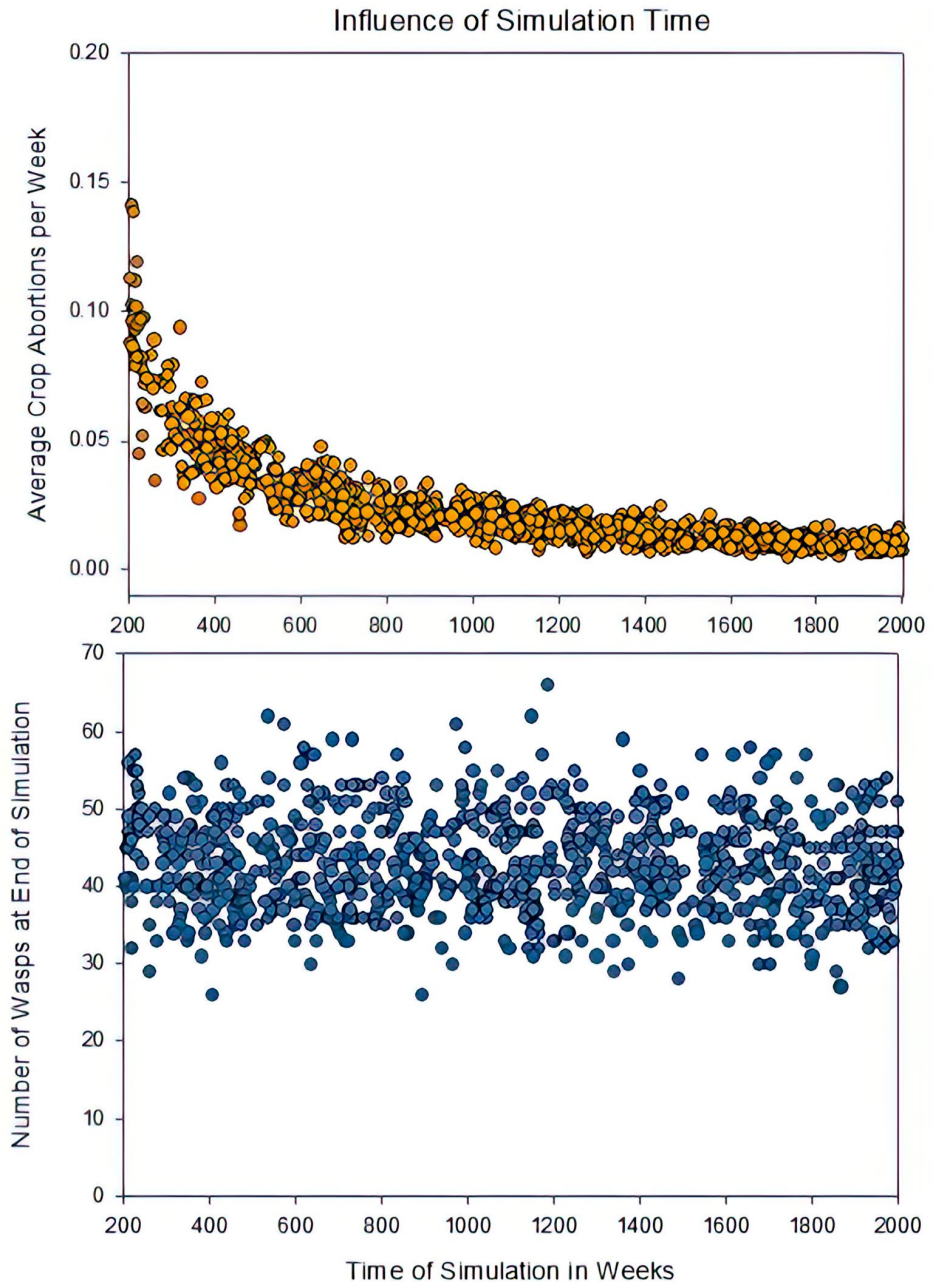
Sensitivity analysis of the influence of simulation time on the number of wasps and crop abortions.

The influence of simulation time was also explored. The model was run for a period ranging from 200 to 2000 weeks (Figure 7). It was found that though the number of wasps at the end of the simulation varied, there was no trend. There was a trend in the number of abortions, with the numbers decreasing exponentially.

**FIGURE 8:**
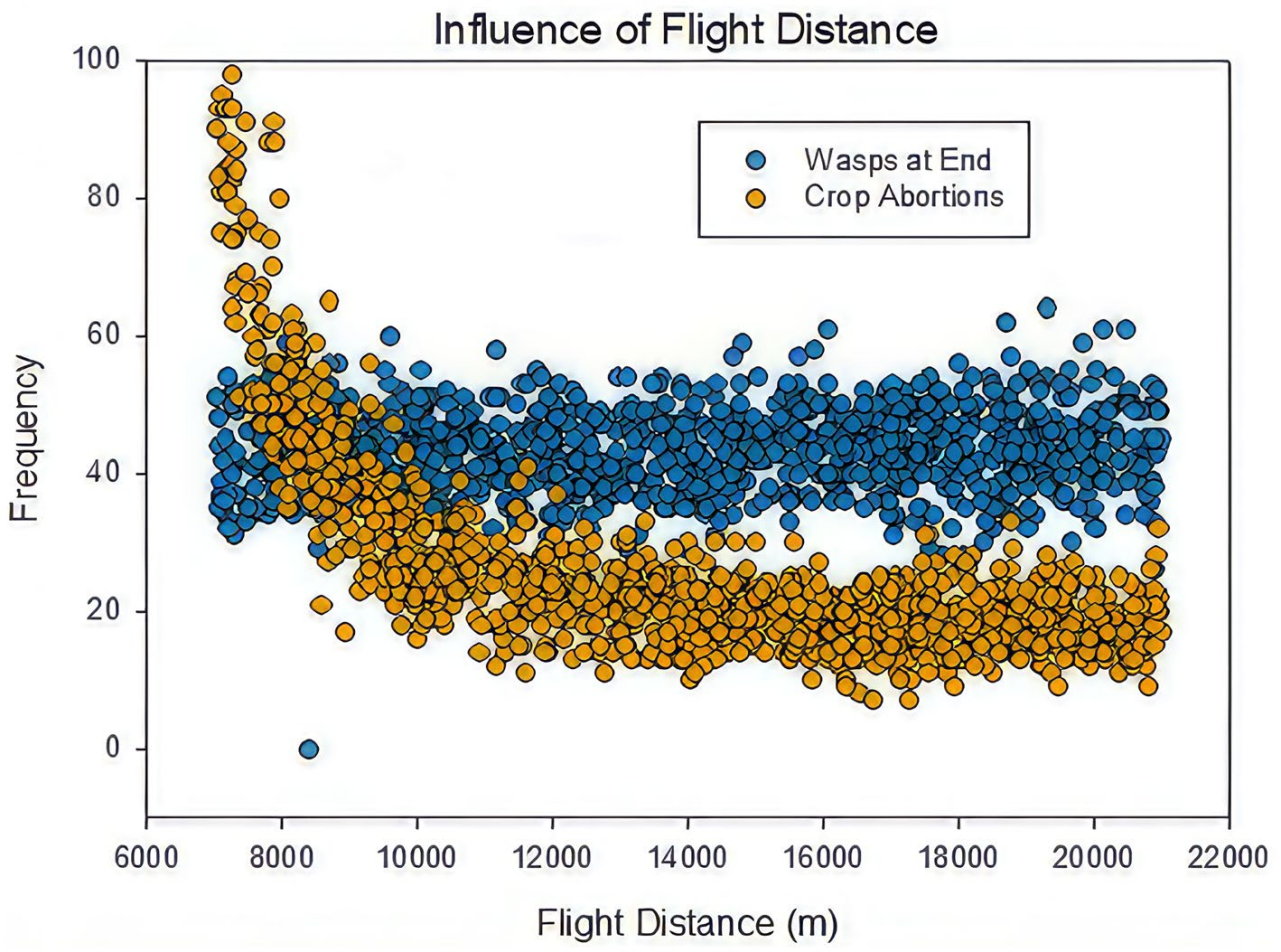
Sensitivity analysis on the influence of flight distance on the number of wasps and crop abortions at the end of the simulation.

A sensitivity analysis was run that varied the distance that wasps were able to fly, ranging from 7.5 to 22.0 km. There was no trend in the number of wasps present at the end of a simulation based on flight distance, but the number of crop abortions decreased to a threshold (Figure 8).

### 3.1 Pollination Network

**FIGURE 9:**
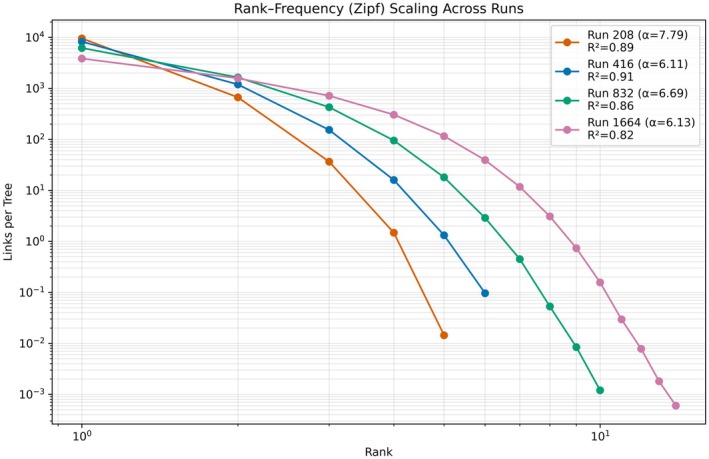
Zipf rank–frequency analysis of wasp connections across simulation runs. Rank–frequency distributions of links per tree (used here as a measure of wasp connectivity) are shown for four simulation runs (208, 416, 832, and 1664 individuals).

A series of model runs were conducted to examine the number of links based on the number of trees and the length of the simulation (Figure 9). The results indicate that the number of links follows a power law, and the number of links is dictated by the length of the simulation, with more links developing over a longer simulation time. The number of links between individual trees and other trees at a single time step (day), as well as the frequency of those links, follows a normal distribution (Figure S2). In contrast, the frequency with which specific tree pairs are linked follows a power‐law distribution (Newman 2005).

Within each run, trees were ordered by decreasing number of connections, and links per tree were plotted against rank on logarithmic axes. Linear regression was performed in log10–log10 space to estimate the scaling exponent (*α*), calculated as the negative slope of the fitted line; *R* ^2^ values indicate goodness of fit. Across runs, the distributions exhibit approximately linear trends in log–log space over multiple ranks, a pattern consistent with heavy‐tailed behavior and potential power‐law scaling. As run size increases, the upper tail extends and the fit improves modestly, suggesting increasing stability in the hierarchical structure of connectivity.

## 4 Discussion

A spatially explicit simulation model was developed to investigate the critical population size (CPS) required for the mutual persistence of *Ficus* trees and their obligate fig wasp pollinators. The CPS threshold was defined as the boundary between parameter settings that consistently led to extinction—where no wasps remained at the end of any simulation run—and those that supported persistence, in which at least one tree retained wasps at the end of every run. This transition represents a clear shift between regimes of population failure and long‐term persistence under the modeled conditions.

The model is defined as an agent‐based model (ABM) because individual trees make decisions on flowering based on information received from wasps, and all interactions can be recorded, allowing the links and connections between trees to be examined. In this way, the genetics or pollination history of trees could be analyzed. In addition, a sub‐model of wasp breeding, heredity, and competition could be employed. Developing an ABM allows for information and attributes for each tree to be saved and applied in the trees' decision making process. These attributes can be changed for an individual throughout a simulation.

A sensitivity analysis for each scenario was conducted to explore the CPS necessary for the persistence of wasp populations in this spatially explicit model (49,000 model runs for each scenario). The model, which incorporates landscape structure and dispersal distance, allowed for the examination of spatial dynamics and their influence on wasp movement and population stability. Results from scenarios suggest that wasps capable of flying longer distances are more resilient to local extinctions, as they can effectively recolonize areas over time. This capacity for spatial recolonization is particularly important in systems with multiple, asynchronously fruiting tree populations, where aborted crops may facilitate the temporal resynchronization of reproductive cycles across the landscape. While the model was developed specifically for a fig–wasp system, it is generalizable and could be applied to other insect‐mediated plant pollination interactions where spatial dynamics and dispersal are critical components of ecological stability. For example, the spatial dimension could be collapsed to show pollination of an individual flower with trees now considered pistil and stamen. The model could also be modified to develop different pollination strategies.

Based on flowering phenology data from *Ficus natalensis* (Bronstein et al. 1990) and wasp flight distances associated with *Ficus dugandii* (Nason et al. 1998), the CPS was estimated to be approximately 245 trees. This is higher than the CPS calculated in Bronstein et al. (1990). This is likely due to a different way to determine CPS, with our use of the minimal number of wasps present at the end of the simulation and a statistical decision tree approach to determine CPS. Also, our model was run 49,000 times for each scenario. The model results indicated that both the density of *Ficus* trees and the flight distance of pollinating wasps are essential for maintaining stable populations. At each time step, the number of connections between an individual tree and neighboring trees followed a normal distribution, reflecting temporal variation in flowering and dispersal opportunities (Figure S2). However, when assessed over the duration of an entire simulation, the number of links per tree exhibited a scale‐free distribution, suggesting that certain trees function as connectivity hubs within the pollination network (Figure 8). The model also incorporated the ability of trees to resynchronize flowering cycles through shortened reproductive phases triggered by the absence of pollinators, resulting in aborted fruit crops. This mechanism allowed asynchronous populations to regain synchrony over time, contributing to the resilience and stability of the fig–wasp mutualism across spatially distributed landscapes.

Differences between the estimated critical population size (CPS) in this study and those reported by Bronstein et al. (1990) and Kameyama et al. (1999) can be attributed to differences in initial parameterization, CPS calculation methods, simulation duration, and model structure. The model presented here incorporates spatial dynamics and explicitly accounts for wasp flight distance. It defines CPS as the complete absence of wasps at the end of any simulation run, rather than using a persistence threshold across runs. Additionally, the model records all interactions among trees, enabling detailed analysis of landscape connectivity and its influence on population persistence.

Similar to Kameyama et al. (1999), this study found that the persistence period of the wasp population increases exponentially with the number of trees, corresponding to a transition phase observed in our model runs. Additionally, the model revealed that as the number of trees increases, crop abortion eventually stabilizes, and longer simulation durations result in reduced abortion rates—suggesting that asynchronous flowering becomes more effective over time and highlighting the resilience and self‐organizing capacity of the fig‐wasp pollination network.

Density and wasp flight distance are two parameters that allow for the network of *Ficus* asynchronous flowering to be maintained. Since wasp flight distance and *Ficus* flowering phenology vary for each mutualistic association, knowledge of each is vital in designing management plans in highly fragmented forests (Mawdsley et al. 1998). The model presented in this paper is flexible and can be run with new parameterization to examine other *Ficus* species and even other tropical trees and pollinators (Molbo et al. 2003). Multiple wasp species associated with a *Ficus* species and wasp species utilizing multiple *Ficus* species further develop the complexity of such pollination networks and critical population size estimates. Modeling such complex network behavior is highly beneficial in understanding *Ficus* pollination in a theoretical framework. The model presented in this paper could easily be modified to explore multiple pollinating wasp species or a wasp species to pollinate multiple *Ficus* species.

The number of links between a tree and other trees, and their frequency follows a normal distribution (Figure S2). However, the number of times specific trees link follows a power law. This suggests that localized trees begin to asynchronously sync their flowering patterns to maximize the ability to maintain the pollinating wasp population and/or possibly increase their pollinating partners. This suggests a more resilient pollinator system. A few trees link to each other more frequently, indicating that these trees are important hubs or nodes in maintaining the wasp population.

Across both standard (4‐year) and extended (40‐year) simulations, model outcomes were consistent, indicating that initial conditions did not materially influence CPS estimates once the system had equilibrated. When the model was run longer than 208 weeks (4 years), trees developed more links. Trees were not added or removed over the 2080 weeks (40 years) of simulation. The number of links in this network is scale‐free. This suggests that specific trees are hubs and crucial in maintaining the wasp population. This also suggests that localized trees begin to synchronize their local asynchronous flowering sequence.

**FIGURE 10:**
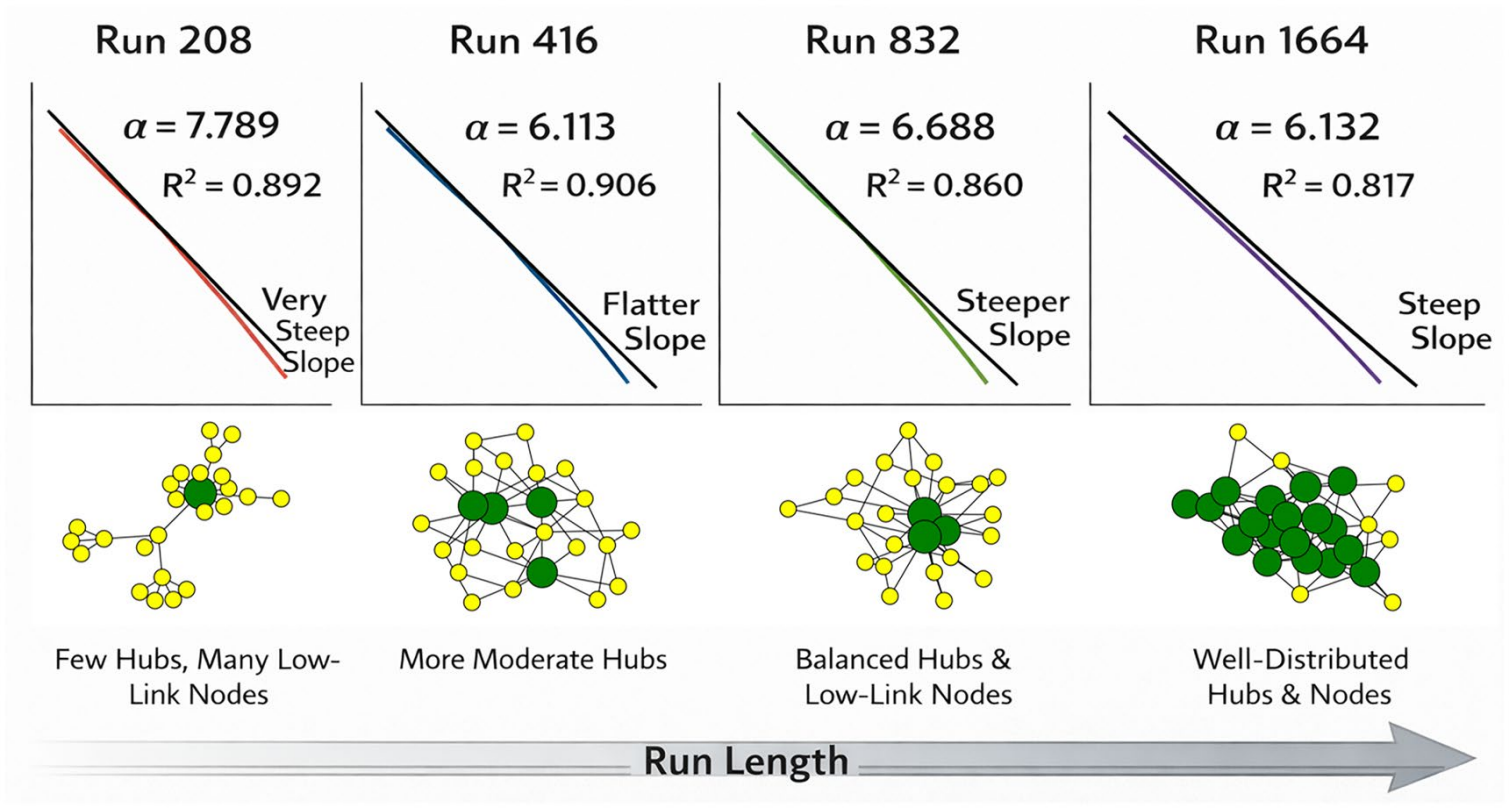
Schematic of the model simulation length in weeks (208, 416, 832, 1664), scaling exponent (*α*), and *R* ^2^, along with an example of what a network would look like for these different networks.

The rank–frequency distributions of wasp connections exhibited approximately linear relationships in log–log space across simulation runs, a pattern consistent with heavy‐tailed structure and potential Zipf‐like scaling. This suggests that connectivity is unevenly distributed, with a small number of highly connected trees and a larger number of weakly connected ones (Figure 10). As population size increased, the upper tail of the distribution extended and the linear fit improved modestly, indicating that hierarchical structure in connectivity becomes more apparent at larger system sizes. Importantly, while these patterns are consistent with power‐law behavior, they should not be interpreted as definitive evidence of strict power‐law scaling. Rather, they indicate that wasp–tree interactions generate disproportionately influential nodes within the network, a structural feature that may have implications for stability, persistence, and estimates of critical population size. Trees that act as hubs are important in this temporal sequence and may connect populations of *Ficus* that are spatially separated (small world). With forest fragmentation occurring, these hubs may prove vital in the maintenance of *Ficus* populations. Further work on these subjects would be possible to examine with this model.

### 4.1 Model Limitations

This study presents a theoretical, spatially explicit modeling framework designed to explore mechanisms underlying persistence in fig–fig wasp mutualisms. Because the model is theoretical and not intended to represent a specific fig–wasp species pair, results should not be interpreted as quantitative predictions for any single natural system. Instead, the parameter combination is used to illustrate general conditions under which persistence or extinction dynamics emerge. While fig–wasp systems exhibit substantial variation in key traits such as phenology and dispersal, many systems remain incompletely parameterized in the empirical literature. In this context, this model provides not only a conceptual framework for understanding persistence dynamics, but also a guide for identifying which parameters are most critical to measure in specific fig–wasp systems to improve future empirical and modeling efforts. As such, the results should not be interpreted as universally applicable to all fig–fig wasp systems. Flowering phenology, reproductive synchrony, wasp flight capacity, mortality rates, multiple pollinating species, and demographic structure vary substantially among fig species and across ecological conditions (Cerezini et al. 2024; Yu et al. 2022, 2021; Suleman et al. 2014). This model does not include the influence of seasonality or the distribution of trees on the landscape (Pothasin et al. 2016; Zhang et al. 2014). The parameter values and scenario configurations explored here represent a defined subset of biologically plausible conditions rather than the full diversity of natural systems.

In addition, field‐based or empirical data on species‐specific flowering variance, dispersal distances, and interaction networks remain limited for many fig–fig wasp systems. Consequently, while the model identifies mechanisms and thresholds that emerge under assumptions, quantitative predictions should be interpreted cautiously until validated with detailed empirical observations. Future work incorporating system‐specific data will be essential for refining parameterization and evaluating the generality of the patterns observed here.

Figs often exhibit pronounced dichogamy, with distinct male and female floral phases occurring sequentially within individual trees. In this theoretical model in this paper, the model represents flowering as a single interval while maintaining separate male and female phases within that interval. However, this model framework is flexible and could collapse the temporal separation, enabling male and female phases to be treated as co‐occurring on a tree within the same time.

We also acknowledge that the model employs a simplified representation of wasp‐mediated pollination. A single surviving wasp is considered sufficient for a successful interaction, and network links are established in a binary manner regardless of wasp abundance. While this captures the presence of interactions, it does not account for variation in wasp numbers, which can influence pollination success and the strength of network connections (Yu et al. 2021; Sun and Wang 2019). As a result, estimates of CPS may be biased by this simplification. The ecological realism of these interactions is limited. Incorporating abundance‐dependent interactions in future work would provide a more accurate representation of pollination dynamics.

The model assumes that a pollination link is established whenever at least one wasp survives, treating pollination as a threshold (presence–absence) process rather than a continuous function of pollinator abundance. This threshold assumption may influence CPS estimates by potentially overestimating persistence, as it does not account for reduced pollination probability at low wasp abundances. By treating pollination as a presence–absence process rather than a continuous function of pollinator abundance, the resulting effects on CPS cannot be determined within the current model structure, since the relationship between pollinator abundance and effective pollination is not explicitly modeled.

Tree positions are generated through random placement across the landscape, without incorporating explicit spatial structure such as habitat patches, dispersal corridors, or edge effects. While this omission precludes direct representation of specific fragmentation geometries, the model is intended as a general theoretical exploration of how spatial distribution influences persistence rather than a spatially explicit prediction for any specific landscape. The use of random tree placement across a large ensemble of simulations is therefore intended to sample a wide range of potential spatial configurations, providing insight into the general persistence of wasp populations under unstructured spatial heterogeneity. Incorporating explicit landscape structure represents an important extension for future work, particularly for evaluating CPS under defined fragmentation scenarios.

Despite these limitations, the framework offers several novel contributions. Unlike many previous models of fig–fig wasp mutualism, this approach explicitly incorporates spatial structure and allows individual trees to be represented as discrete entities within a network. This enables the tracking of all pollination interactions among trees over time and permits direct examination of how spatial configuration, flowering asynchrony, and dispersal constraints jointly influence persistence dynamics. By integrating spatial processes with interaction networks, the model provides a new perspective for investigating the coupled ecological dynamics of figs and their pollinating wasps.

## 5 Conclusion

In conclusion, this study demonstrates that both tree density and wasp flight distance are key parameters for sustaining fig‐wasp mutualisms, particularly in fragmented landscapes in which spatial and temporal dynamics shape pollination success. The critical population size (CPS) was evaluated through a sensitivity analysis of tree abundance. Wasp flight distance plays a key role in determining sustainable *Ficus* populations. Additional analyses explored the influence of other model parameters and how the system's dynamics might shift over longer simulation periods. The emergence of scale‐free network structures highlights the importance of individual trees in maintaining connectivity and synchrony across populations. These findings underscore the value of spatially explicit modeling for understanding and managing complex plant‐pollinator systems and offer a flexible framework for exploring critical population thresholds across diverse *Ficus*‐wasp associations. This model develops a new concept on what is deemed a viable pollinating population and includes new spatial attributes and the ability to track individual trees and wasps.

## Author Contributions

**Michael Palace:** conceptualization (equal), data curation (equal), formal analysis (equal), funding acquisition (equal), investigation (equal), methodology (equal), project administration (equal), resources (equal), software (equal), supervision (equal), validation (equal), visualization (equal), writing – original draft (equal), writing – review and editing (equal).

## Funding

Funding was provided by NASA's ROSES Interdisciplinary Research in Earth Science (IDS) Program through award 80NSSC20K1266 (Smoke on the Water: Lake‐based Calibration of Amazonian Fire Histories).

## Conflicts of Interest

The author declares no conflicts of interest.

## Supporting information


**Figure S1:** Schematic of the pollination network. A model run with 3000 trees for a time period of 2080 weeks. Node size is based on the number of links for that tree. Links between trees are colored on the number of links between two trees.
**Figure S2:** Number of links in the simulations run with a different number of trees and for different lengths of time. Model runs include different numbers of trees and different lengths of time of simulations. Each simulation was run 100 times and average values are reported.

## Data Availability

All data supporting the findings of this study are available at the online data archive site found in the reference below. The code used in the analysis is also provided in the link below. This includes python code for network figure and Zipf analysis. Palace, Michael, 2025, “Spatially Explicit Agent‐Based Model Simulating *Ficus* Trees and their Obligate Wasp Pollinator”, https://doi.org/10.7910/DVN/C2CLYA, Harvard Dataverse, V3.
