# Supplementary material for: Asynchronous Synchronization: A Spatially Explicit Agent‐Based Model Simulating Ficus Trees and Their Obligate Wasp Pollinator
Source: Ecol Evol. 2026 Jun 10;16(6):e73778. doi: 10.1002/ece3.73778 (PMC13250636; doi:10.1002/ece3.73778)
Supplement: Supplementary file 1 — Figure S1: Schematic of the pollination network. A model run with 3000 trees for a time period of 2080 weeks. Node size is based on the number of links for that tree. Links between trees are colored on the number of links between two trees. Figure S2: Number of links in the simulations run with a different number of trees and for different lengths of time. Model runs include different numbers of trees and different lengths of time of simulations. Each simulation was run 100 times and average values are reported. [file ECE3-16-e73778-s001.docx]

Supplemental Material


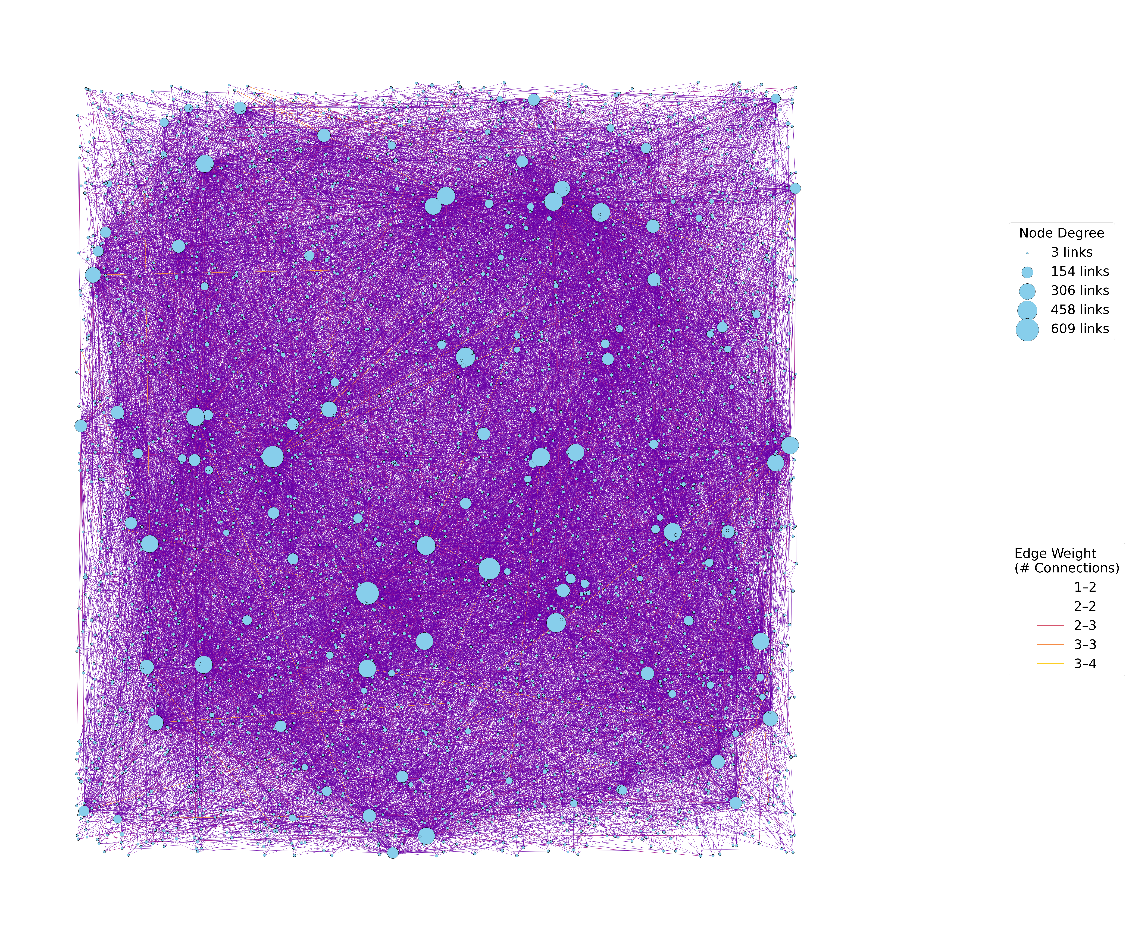


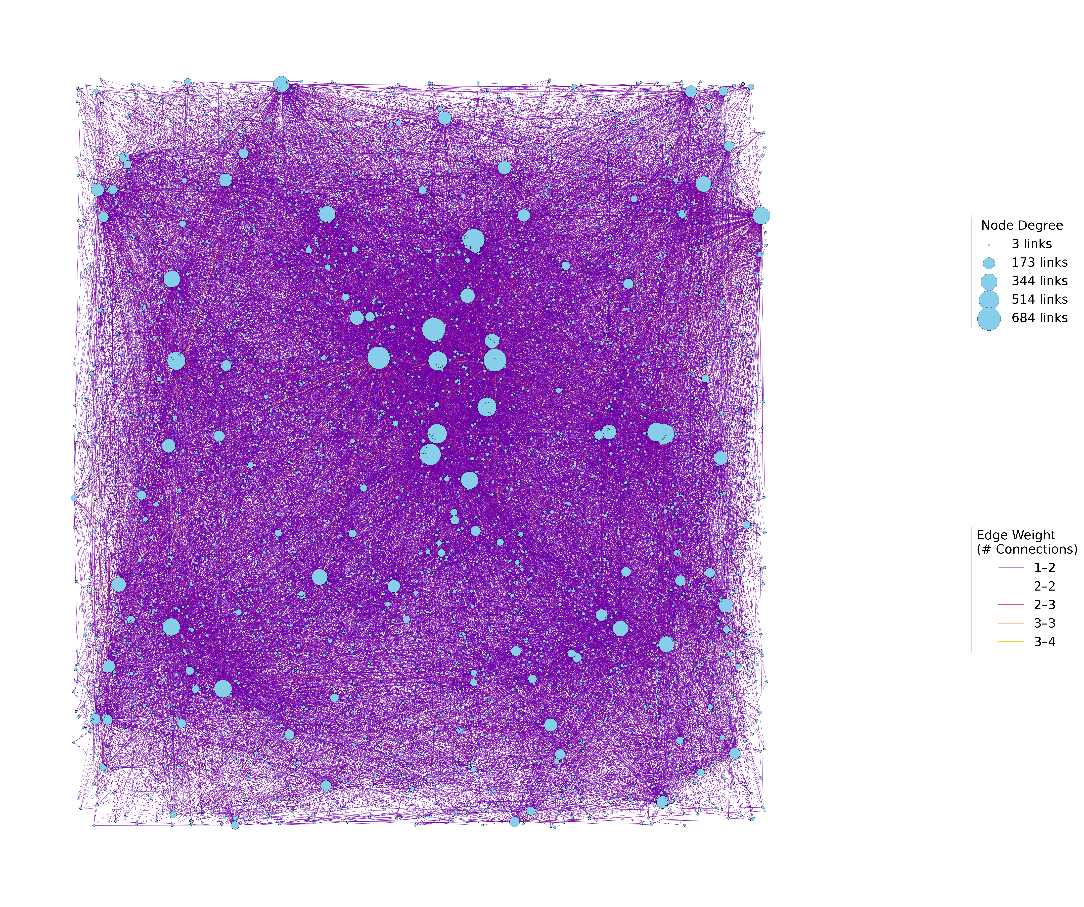


Supplemental Figure 1. Schematic of the pollination network. A model run with 3000 trees for a time period of 2080 weeks. Node size is based on the number of links for that tree. Links between trees are colored on the number of links between two trees.


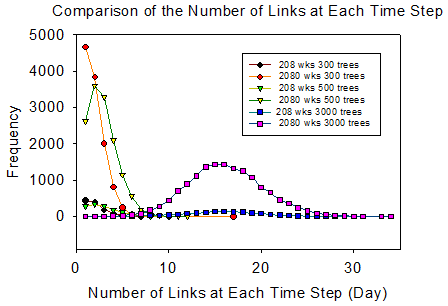


Supplemental Figure 2. Number of links in the simulations run with a different number of trees and for different lengths of time. Model runs include different numbers of trees and different lengths of time of simulations. Each simulation was run 100 times and average values are reported.
